# The impact of T cell percentage at CTL019 T cell expansion process initiation on post-harvest T cell composition.

**DOI:** 10.1186/2051-1426-3-S2-P16

**Published:** 2015-11-04

**Authors:** Tatiana Golovina, Eric Drew, Joseph Sieling, Brian Gismonde, Marty Giedlin

**Affiliations:** 1 Novartis Pharmaceuticals, Morris Plains, NJ, USA

## Study objectives

Assess the range of starting T cell percentage in the CTL019 expansion process.

## Results

The current CTL019 process targets 450e9 total nucleated cells (TNC) at seeding of the static culture bags prior to activation and transduction (Kalos M et al. 2011; Maude et al. 2014). However, less is known on the impact of a range of T cell numbers within the TNC population on, transduction efficiency and expansion. Healthy donor T cells cultures were evaluated in the range of the starting T cell percentages between 1.7% and 97.7%. T cells expanded in all tested conditions with the population doubling range between 3.7 and 8.5, and the percentages of T cells at the end of the culture period were 94.9 ± 5.0%. The major non-target cell population at the end of culture was NK cell population (4.8 % ± 2.9%).

The feasibility of expanding T cells from leukemic donors with very low T cell content was assessed on three ALL patient samples with original T cell contents of 1.4%, 1.2% and 2.0%. After polyclonal stimulation and growth in culture T cell percentages were respectively the following: 78.1%, 95.9% and 1.1% (non-expander), supporting the potential of some ALL patients with low T cell counts to meet cell number requirements after 9 day expansion for cell therapy in the absence of T cell enrichment steps.

## Conclusions

Healthy donor T cells can be used to assess the impact of low percentage T cell number in the CTL019 manufacturing process. These studies support the use of low T cell percentages as starting material for the CTL019 process. However, the impact on the final product, e.g. transduction efficiency, has to be further evaluated. Finally, in case of the ALL patients, the outcome can be different between donors.

